# 

**Published:** 1848-07

**Authors:** 


The next number of this Journal will contain a report on Cholera, by Prof. C. B. Coventry; and the number for September an article by Prof. C. A. Lee.

*Delegates from Buffalo to the American Medical Association.*—We omitted heretofore to mention the names of the delegates from this city who attended the late meeting of the American Medical Association. Buffalo was represented by three delegates. Dr. Bryant Burwell, attended as delegate from the Erie County Medical Society. He was also one of the delegates appointed by the State Society. Dr. Walter Cary, was the delegate for the Buffalo Medical Association; and Dr. F. H. Hamilton from the Medical College. We hope soon to receive the printed proceedings and reports.

